# Real world observation of fatigue in multiple sclerosis comparing self-guided online cognitive behavioral therapy versus psychoeducation

**DOI:** 10.1177/20552173261468118

**Published:** 2026-08-25

**Authors:** Maria Protopapa, Jana Samarin, Muriel Schraad, Livia S Lang, Marianne Hahn, Frauke Zipp, Stefan Bittner

**Affiliations:** 1 Department of Neurology, Research Center for Immunotherapy (FZI) and Focus Program Translational Neuroscience (FTN), Rhine-Main Neuroscience Network (RMN2), 39068 University Medical Center of the Johannes Gutenberg University Mainz, Mainz, Germany

**Keywords:** Multiple sclerosis, fatigue, cognitive behavioral therapy, digital health, psychoeducation

## Abstract

**Background:**

Fatigue is a common symptom in people with multiple sclerosis (pwMS) with severe implications. A self-guided, internet-based intervention to reduce fatigue in MS, based on cognitive behavioral therapy (CBT), is approved as a digital health application (DiGA) in the German healthcare system. However, real-world evidence for efficacy is lacking.

**Methods:**

Of the 165 patients screened for fatigue using the Fatigue Scale for Motor and Cognitive Functions (FSMC), 129 were diagnosed with fatigue (FSMC ≥ 43) and enrolled. Patients with fatigue were randomized 1:1 to either a 12-week CBT-based online intervention (elevida, a CE-certified, self-guided DiGA for the management of MS-related fatigue; n = 64) or a psychoeducation arm (n = 65). After 12 weeks, patients in the psychoeducation group could cross-over to the CBT-based online intervention group. The primary outcome was FSMC-change.

**Results:**

The 12-week follow-up showed a reduction in FSMC in the primary CBT-based online intervention group (repeated measures ANOVA: F(1,48) = 10.99, p = 0.002), as well as the crossover-group (repeated measures ANOVA: F(1,15) = 5.81, p = 0.029). However, between-group analyses did not demonstrate superiority of the CBT-based online intervention over psychoeducation (mean difference 1.8 points, 95% CI −2.0 to 5.6; p = 0.35).

**Conclusion:**

Our results highlight CBT-based online interventions as promising supportive tools for managing cognitive fatigue in pwMS.

## Introduction

Fatigue is a highly prevalent symptom in people with multiple sclerosis (pwMS) and other autoimmune diseases and is considered a multidimensional syndrome with somatic-physical, cognitive and psychosocial aspects.^1^ Commonly defined as a lack of physical and/or mental energy that interferes with daily activities,^2^ fatigue can strongly impair quality of life in pwMS. Up to 80% of pwMS describe fatigue as their most disabling symptom and it is one of the most frequent causes for MS-related early retirement.^3^ However, despite extensive research, the exact pathomechanism of MS-related fatigue remains unclear.^4^ Pro-inflammatory cytokines may contribute via microglia activation and effects on the hypothalamus-pituitary axis.^5^ Brain atrophy, especially in white and gray matter,^6^ as well as structural changes in the caudate, pons^7^ and plexus choroideus^8^ have also been linked to fatigue. Moreover, functional magnetic resonance imaging studies suggest a close connection between the cortico-striato-thalamo-cortical loop and fatigue.^9^

In clinical practice, symptoms and impairments related to fatigue are assessed using standardized questionnaires, for example, the Fatigue Scale for Motor and Cognitive Functions (FSMC),^10^ the Fatigue Severity Scale (FSS),^11^ or the Modified Fatigue Impact Scale (MFIS).^12^ FSMC assesses the two main domains of fatigue: cognitive (FSMCc) and motoric (FSMCm). A key advantage is its ease of administration with only 20 items; established cut-off values for the total FSMC score and both subscales allow classification of fatigue severity as mild (43–52 points), moderate (53–62 points), or severe (≥63 points). Assessment of fatigue symptoms in clinical practice is particularly important to differentiate from comorbid conditions causing reduced energy, such as depression, metabolic (e.g., vitamin deficiencies and hypothesis) or sleep disorders.

Managing MS-related fatigue remains a challenge, with non-pharmacological approaches such as cognitive behavioral therapy (CBT)^13,14^ and mindfulness-based intervention,^15^ considered standard-of-care. In terms of pharmacological treatment, however, there is currently no conclusive evidence, as amantadine, modafinil, and methylphenidate have shown no benefit in placebo-controlled, double-blind studies^16^ and are not recommended as standard treatment.

Digital health applications (DiGA) have been recently introduced into the German Health system. Current German MS guidelines recommend the use of DiGA, including a CBT-based program, as part of a multimodal approach to fatigue management.^17^ Specifically, a CE-certified self-guided CBT-based online intervention named elevida (GAIA AG) can now be prescribed to pwMS in clinical routine, at no cost for the patients. Utilizing a simulated dialogue, pwMS receive educational information about fatigue and are given homework tasks. Patients are advised to use elevida once or twice per week. Pöttgen et al.^18^ showed a significantly greater reduction in Chalder Fatigue Scale scores in pwMS using elevida compared to a control group, which consisted of patients on a waiting list for the use of the digital online intervention. However, this prior study was conducted in a highly structured trial setting, used a passive control condition, and assessed fatigue using a global outcome measure. In contrast, the present study evaluated elevida under routine clinical conditions with less structured monitoring and broader real-world conditions. We further compared elevida with an active control condition consisting of standardized, non-interactive psychoeducation rather than a waiting-list control. Finally, we applied the FSMC, allowing separate assessment of cognitive and motor fatigue and thereby extending previous findings beyond global fatigue measures.

Accordingly, the aim of our study was to evaluate the effectiveness of elevida, a CBT-based online intervention, in light of two important knowledge gaps: (i) to assess the efficacy of symptom reduction in pwMS with fatigue in a clinical routine setting; and (ii) to compare efficacy to a control group that received standardized psychoeducation as an active comparator.

## Patients and methods

### Ethics

The study was performed in compliance with the Declaration of Helsinki and was approved by the local ethics committee (Ethikkommission der Landesärztekammer Rheinland Pfalz 2019-14758_1); participants gave written informed consent.

### Study design

**Figure 1.**
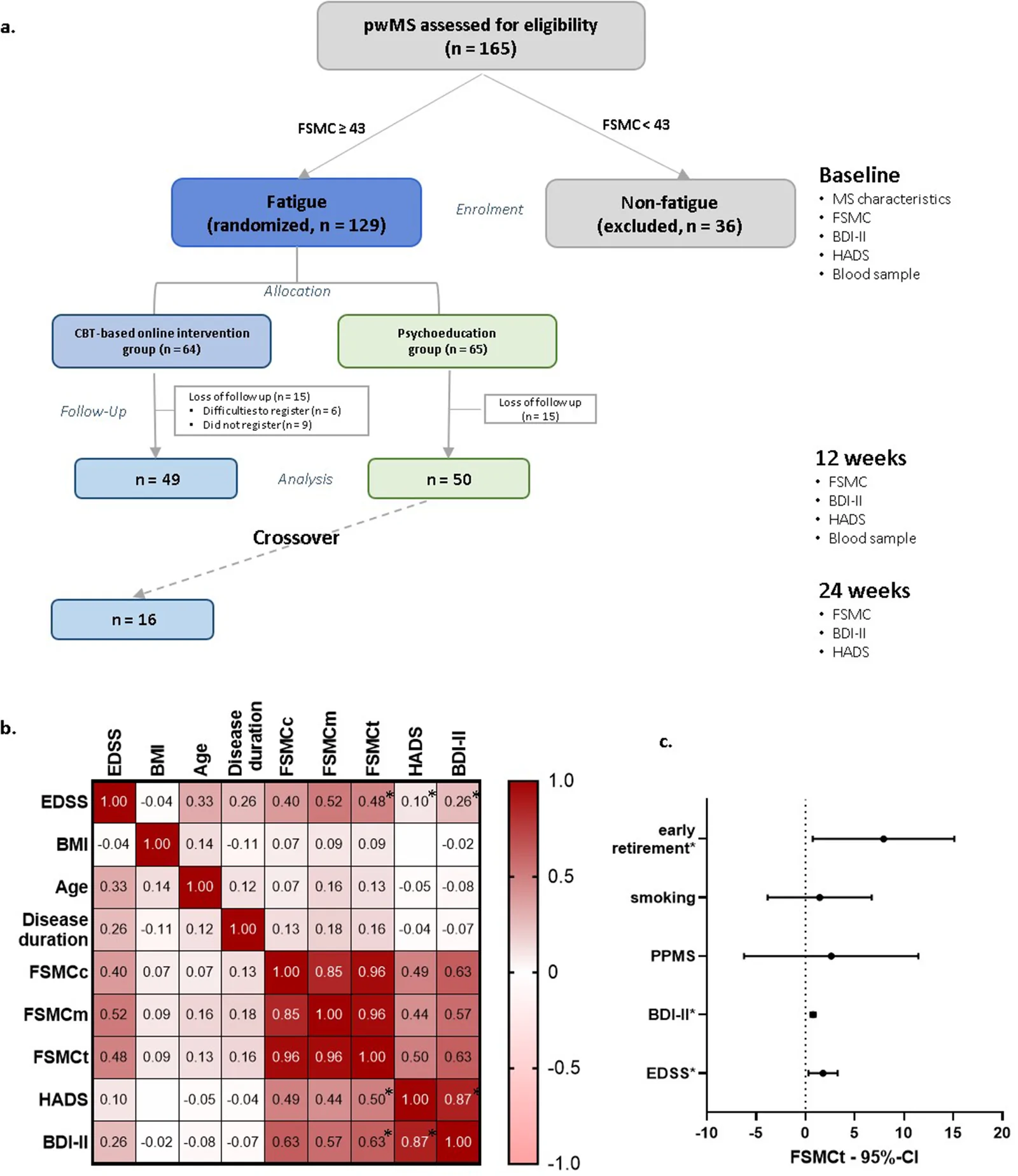
Study design and baseline characteristics. (a) Out of 165 pwMS, 129 had an FSMC ≥ 43 and were randomized into either a CBT-based online intervention (elevida) or psychoeducation group and followed up for 12 weeks. Afterwards, participants in the psychoeducation group had the option to switch to the CBT-based online intervention group. Dropouts and assessed parameters are indicated in the figure. (b) Correlation matrix showing the correlation coefficients of the associations between FSMCc, FSMCm, FSMCt and clinical baseline parameters. Pearson's r is additionally translated into a color scale ranging from light red (r = −1) to deep red (r = + 1). Correlations with p < 0.05 were considered statistically significant and are marked with *. (c) Forest plot showing the determined regression coefficient for FSMCt. The multiple linear regression model revealed depression, EDSS and early retirement as significant predictors for the dependent variable FSMCt. BDI-II: Beck Depression Inventory II; BMI: body mass index; CI: confidence interval; EDSS: Expanded Disability Status Scale; FSMCc: Fatigue Scale for Motor and Cognitive Functions cognitive; FSMCm: Fatigue Scale for Motor and Cognitive Functions motoric; FSMCt: Fatigue Scale for Motor and Cognitive Functions total; HADS: Hospital Anxiety and Depression Scale.

PwMS visiting the outpatient clinic of the Department of Neurology at the University Medical Center Mainz from February 2023 to February 2024 were screened for fatigue as part of routine clinical care using the FSMC (n = 165). Eligible patients (FSMC ≥43, age ≥18 years, confirmed MS diagnosis according to McDonald-Criteria 2017, no relapse or steroid treatment within the preceding 3 months, no acute psychiatric disorder and the ability to use the CBT-based online intervention) (n = 129) were randomized 1:1 to a 12-week CBT-based online intervention (elevida) (n = 64) or standardized psychoeducation (n = 65) (Figure 1(a)).

The study was conducted as a pragmatic randomized controlled trial included in routine outpatient care and was not prospectively registered. Randomization was computer-generated; due to the nature of the intervention, blinding was not feasible.

Baseline assessments included FSMC, Hospital Anxiety and Depression Scale (HADS), and the Beck Depression Inventory-II (BDI-II). All outcomes were collected using self-administered questionnaires. Additional clinical and demographic data included age, sex, MS type, disease duration, Expanded Disability Status Scale (EDSS), and medication. In a self-reporting assessment, patients were asked about their active work status, smoking-, alcohol-, sport-, and diet-habits and any previous psychotherapeutic experience.

At week 12, FSMC, HADS, and BDI-II were reassessed in both groups. Patients in the psychoeducation group then had the option to cross-over to the CBT-based online intervention group (n = 16) and underwent an additional assessment at week 24.

### CBT-based online intervention arm (elevida)

Elevida (GAIA AG) is a CE-certified, self-guided online intervention based on CBT principles, which can be prescribed as part of the clinical routine to pwMS with fatigue in the German Healthcare system.^18^ The program is structured into sequential modules of psychoeducation about fatigue, cognitive restructuring, behavioral activation, stress and energy management, and sleep hygiene. Patients engage in a ‘simulated dialogue’, answer multiple-choice questions, and access homework tasks and techniques. Patients visited our outpatient clinic and received a prescription for the program to submit to their health insurance. Patients then received an activation code to register on the platform. Patients were advised to access the program once to twice per week. Usage of the CBT-based online intervention was assessed retrospectively using a self-report questionnaire capturing frequency and duration of use. A session duration of ≥30 min per week was used as an exploratory indicator of higher engagement.

### Psychoeducation arm

Psychoeducation is an intervention with systematic, structured, and didactic knowledge transfer for an illness and its treatment.^19^ The aim is to enable patients to cope with the illness and to improve its treatment adherence and efficacy. Psychoeducation can generally be useful in the management of chronic diseases.^20^ In our study, the psychoeducation control condition consisted of a written, non-interactive program specifically developed for this study that focused on MS-related fatigue. The material (available in both printed and digital formats, without interactive components or behavioral exercises) consisted of two parts, information on the (i) definition, diagnosis, and treatment of fatigue; and (ii) pathogenesis of fatigue. Engagement with the materials was self-directed, with no predefined duration or structured guidance; no objective or quantitative measures of engagement were collected. Representative samples of the psychoeducation materials in the original German language are provided as Supplementary Material.

### Outcome measures

Outcome measures were assessed at two predefined time points: baseline (prior to randomization) and 12-week follow up. The primary outcome measure was the FSMC.^10^ Although no universally established Minimal Clinically Important Difference (MCID) exists for the FSMC, prior studies in pwMS have suggested that a reduction of approximately 9–10 points in the FSMC total score may represent a clinically meaningful improvement.^21^ Secondary outcomes (BDI-II, HADS) were assessed at the same time points. All outcomes were collected using self-administrated questionnaires completed by participants either during routine outpatient visits or remotely.

### Statistical analysis

The statistical analyses were conducted using RStudio version 1.2.1335 with R version 4.3.2, SPSS version 29.0.0.0 (IBM Corp., USA), and Graphpad Prism version 9.5.1. Normality was assessed using Shapiro-Wilk tests. Group comparisons were assessed using the t-test or one-way ANOVA for normally distributed variables, and the Mann-Whitney U or Kruskal-Wallis test for non-normally distributed variables. Categorical variables were compared using the Chi-square or Fisher-exact tests as appropriate.

At baseline, associations between FSMC total score and clinical demographics and psychosocial variables were examined using correlation analyses and group comparisons. Variables at baseline showing significant univariate associations were included in a multiple linear regression model with FSMC total score as the dependent variable to identify factors independently associated with fatigue severity.

Primary analyses were conducted as complete-case analyses and therefore followed a per-protocol approach. An intention-to-treat analysis was not feasible because FSMC follow-up data were unavailable for participants who did not complete the 12-week assessment. Missing data were not imputed. Sensitivity analyses included analyses of participants who crossed over to the CBT-based intervention after 12 weeks and pooled analyses combining the CBT-based intervention and crossover groups; these analyses were exploratory.

Between-group treatment effects on fatigue, depressive, and anxiety symptoms were examined using ANCOVA models with FSMC, BDI-II, or HADS total score at 12 weeks as the dependent variable, treatment group as the independent variable, and the corresponding baseline score as a covariate. Within-group changes over time (baseline versus 12 weeks) were assessed using repeated-measures ANOVA separately for each group. Exploratory analyses to identify characteristics associated with greater benefit from the CBT-based intervention were conducted using multiple linear regression models within the CBT-based intervention group. Change in FSMC total score from baseline to 12 weeks served as the dependent variable, with baseline cognitive and motor fatigue (FSMCc, FSMCm), baseline depressive symptoms (BDI-II), intervention use (frequency and duration), and prior behavioral therapy experience as independent variables.

Data are shown as the mean ± standard error of the mean (SEM) unless otherwise indicated. P values < 0.05 were considered statistically significant.

## Results

### Baseline comparison of study groups

A total of 165 pwMS were screened for fatigue at the outpatient clinic of the Department of Neurology at the University Medical Center Mainz. Of these, 129 had a FSMC score ≥43 and were diagnosed with fatigue (median age 41.0 interquartile range [IQR 32.0–51.5], 81.4% female). The remaining 36 pwMS did not have fatigue symptoms at the screening time point (median age 45.0 [34.0–51.0], 66.7% female) (Figure 1). Baseline demographic and clinical characteristics are presented in Tables 1 and 2. Overall, groups were well balanced with respect to age, sex, disease characteristics, disability status, fatigue severity, and affective symptoms.

**Table 1.** Baseline data fatigue versus no fatigue.

	Fatigue cohort (n = 129)	No fatigue cohort (n = 36)	P-Value
Sex (female)	105 (81.4%)	24 (66.7%)	0.05
BMI (median[IQR])	24.9 [22.6–31.0]	25.8 [21.0–27.8]	0.48
Age at baseline	41.0 [32.0–51.5]	45.0 [34.0–51.0]	0.41
Diagnosis			
- RRMS	108 (83.7%)	36 (100%)	0.004
- PPMS	12 (9.3%)	0 (0%)	0.04
- SPMS	9 (7%)	0 (0%)	0.10
Age at diagnosis (years)	32.0 [24.5–41.0]	39.5 [28.2–44.5]	0.04
Disease duration (years)	6 [2.00–12.00]	4 [2.25–6.00]	0.09
Disease-modifying treatment			
- No treatment	17 (13.2%)	7 (19.4%)	0.24
- Low efficacy	10 (7.8%)	12 (33.3%)	<0.001
- Intermediate efficacy	39 (30.2%)	13 (36.1%)	0.32
- High efficacy	62 (48.1%)	4 (11.1%)	<0.001
EDSS at baseline	2 [1.0–3.5]	1 [1.0–1.6]	<0.001
FSMC at baseline			
- FSMCt	76.0 [65.5–85.0]	24.0 [20.2–34.7]	<0.001
- FSMCc	38.0 [33.0–42.0]	12.0 [10.0–16.0]	<0.001
- FSMCm	39.0 [34.0–43.0]	12.0 [10.0–19.0]	<0.001
HADS at baseline	13.0 [8.0–19.0]	6.0 [4.0–9.0]	<0.001
BDI-II at baseline	16.0 [10.0–25.0]	2.0 [0.0–5.0]	0.01

BDI-II, Beck Depression Inventory II; BMI, body mass index; EDSS, Expanded Disability Status Scale; HADS, Hospital Anxiety and Depression Scale; FSMC, Fatigue Scale for Motor and Cognitive Functions cognitive; FSMCc, Fatigue Scale for Motor and Cognitive Functions cognitive; FSMCm, Fatigue Scale for Motor and Cognitive Functions motoric; FSMCt, Fatigue Scale for Motor and Cognitive Functions total; PPMS, primary progressive multiple sclerosis; RRMS, relapsing-remitting multiple sclerosis; SPMS, secondary progressive multiple sclerosis.

**Table 2.** Comparison of characteristics for complete fatigue cohort and the CBT-based online intervention subgroup versus psychoeducation subgroup.

	Fatigue cohort (n = 129)	CBT-based online intervention (n = 64)	Psychoeducation (n = 65)	P-Value
Sex (female)	105 (81.4%)	55 (85.9%)	50 (76.9%)	0.26
BMI (median[IQR])	24.9 [22.6–31.0]	24.1 [21.2–30.8]	25.7 [23.1–31.6]	0.23
Age at baseline	41.0 [32.0–51.5]	38.5 [31.0–50.5]	45.0 [35.0–52.0]	0.10
Diagnosis				
- RRMS	108 (83.7%)	52 (81.3%)	56 (86.2%)	0.48
- PPMS	12 (9.3%)	6 (9.4%)	6 (9.2%)	1.0
- SPMS	9 (7.0%)	6 (9.4%)	3 (4.6%)	0.32
Age at diagnosis	32.0 [24.5–41.0]	29.5 [24.0–40.0]	34.0 [25.0–44.0]	0.14
Disease duration	6.0 [2.0–12.0]	6.0 [2.0–12.0]	6.0 [2.0-.12.0]	0.91
Disease-modifying treatment				
- No treatment	17 (13.2%)	5 (7.8%)	12 (18.5%)	0.12
- Low efficacy	10 (7.8%)	4 (6.3%)	6 (9.2%)	0.74
- Intermediate efficacy	39 (30.2%)	19 (29.7%)	20 (30.8%)	1.0
- High efficacy	62 (48.1%)	36 (56.3%)	26 (40.0%)	0.08
EDSS at baseline	2.0 [1.0–3.5]	2.0 [1.0–3.5]	2.0 [1.0–3.9]	0.84
FSMC at baseline				
- FSMCt	76.0 [65.5–85.0]	77.0 [66.3–83.8]	76.0 [64.0–87.0]	0.68
- FSMCc	38.0 [33.0–42.0]	37.5 [32.3–42.0]	38.0 [33.0–42.5]	0.39
- FSMCm	39.0 [34.0–43.0]	39.5 [34.5–43.0]	39.0 [34.0–44.5]	0.96
FSMCt (category)				0.65
- Light fatigue	9 (7.0%)	5 (7.8%)	4 (6.2%)	
- Moderate fatigue	12 (9.3%)	5 (7.8%)	7 (10.8%)	
- Severe fatigue	108 (83.7%)	54 (84.4%)	54 (83.1%)	
FSMCc (category)				0.73
- No fatigue	2 (1.6%)	1 (1.6%)	1 (1.5%)	
- Light fatigue	15 (11.6%)	8 (12.5%)	7 (10.8%)	
- Moderate fatigue	19 (14.7%)	10 (15.6%)	9 (13.8%)	
- Severe fatigue	93 (72.2%)	45 (70.3%)	48 (73.8%)	
FSMCm (category)				0.21
- No fatigue	1 (0.8%)	0 (0%)	1 (1.5%)	
- Light fatigue	15 (11.6%)	6 (9.4%)	9 (13.8%)	
- Moderate fatigue	9 (7.0%)	6 (9.4%)	3 (4.6%)	
- Severe fatigue	104 (80.6%)	52 (81.3%)	52 (80.0%)	
HADS at baseline	13.0 [8.0–19.0]	13.0 [8.0–17.0]	14.0 [8.3–21.0]	0.38
BDI-II at baseline	16.0 [10.0–25.0]	16.0 [10.8–22.0]	16.0 [8.0–27.0]	0.67
Active work status				
- In professional education	8 (6.2%)	3 (4.8%)	5 (7.9%)	0.465
- Full- time employment	46 (35.7%)	19 (30.2%)	27 (42.9%)	0.139
- Part-time employment	33 (25.6%)	23 (36.5%)	10 (15.9%)	0.007
- Retirement	4 (3.2%)	1 (1.6%)	3 (4.8%)	0.309
- Early retirement	23 (17.8%)	12 (19.0%)	11 (17.5%)	0.500
- Homemaker	4 (3.1%)	3 (4.8%)	1 (1.6%)	0.309
- Unknown	10 (7.9%)	4 (6.3%)	6 (9.5%)	0.372
Smoking (yes)	59 (48.4%)	27 (44.3%)	32 (52.5%)	0.234
Alcohol (yes)	87 (71.3%)	44 (72.1%)	43 (14.8%)	0.500
Sports (yes)	67 (55.4%)	33 (55.0%)	34 (55.7%)	1.0
Completed the study	95 (73.6%)	49 (76.6%)	46 (70.8%)	0.550
FSMC at 12wFU				
- FSMCt	76.0 [65.5–85.0]	73.0 [66.0–81.0]	75.5 [62.8–85.0]	0.38
- FSMCc	38.0 [33.0–42.0]	36.0 [31.0–40.0]	37.0 [30.8–43.3]	0.11
- FSMCm	39.0 [34.0–43.0]	37.0 [33.0–43.0]	39.0 [31.0–42.0]	0.91
HADS at 12wFU	12.0 [6.0–19.0]	12.0 [6.3–16.0]	13.0 [6.0–21.3]	
BDI-II at 12wFU	14.5 [9.0–25.0]	14.0 [9.0–18.0]	16.5 [8.75–27.3]	
Found the intervention beneficial	47 (56.0%)	27 (56.3%)	20 (55.6%)	1.0
Fatigue knowledge	57 (64.0%)	34 (66.7%)	23 (60.5%)	0.656
Prior psychotherapeutic session	40 (45.5%)	23 (45.1%)	17 (45.9%)	1.0
Prior behavioral therapy	24 (27.6%)	15 (30.0%)	9 (24.3%)	0.632
Deep psychology therapy	3 (3.4%)	1 (2.0%)	2 (5.4%)	0.572
Psychoanalysis	7 (8.0%)	4 (8.0%)	3 (8.1%)	1.0
Talk therapy	10 (11.4%)	1 (2.0%)	9 (23.7%)	0.002
unknown	2 (2.3%)	1 (2.0%)	1 (2.7%)	1.0
Current use of antidepressant	31 (24%)	17 (26.6%)	14 (21.5%)	0.322

BDI-II, Beck Depression Inventory II; BMI, body mass index; EDSS, Expanded Disability Status Scale; HADS, Hospital Anxiety and Depression Scale; FSMC, Fatigue Scale for Motor and Cognitive Functions; FSMCc, Fatigue Scale for Motor and Cognitive Functions cognitive; FSMCm, Fatigue Scale for Motor and Cognitive Functions motoric; FSMCt, Fatigue Scale for Motor and Cognitive Functions total; PPMS, primary progressive multiple sclerosis; RRMS, relapsing-remitting multiple sclerosis; SPMS, secondary progressive multiple sclerosis.

### Fatigue correlates with disability and depression and the social aspects of fatigue

At baseline, correlations between fatigue severity, clinical characteristics, and psychosocial variables were explored (Figure 1(b); Supplementary Table 1). FSMC total score (FSMCt) correlated moderately with disability (EDSS; r = 0.48, p < 0.001), anxiety (HADS; r = 0.50, p < 0.001), and depressive symptoms (BDI-II; r = 0.63, p < 0.001). FSMCt differed across clinical and social subgroups. Patients with primary progressive MS (PPMS) showed higher fatigue scores than those with relapsing–remitting MS (RRMS: 69.0 [44.7–81.0] versus PPMS: 82.0 [72.0–95.25]; p = 0.006). Higher FSMCt was also observed in smokers compared with non-smokers (77.0 [63.2–85.0] versus 70.0 [54.5–81.0]; p = 0.039) and in patients who were retired or retired early compared with full-time employed individuals (employed full time: 70.0 [52.25–79.0], retired: 91.5 [77.5–98.75], p = 0.02; in early retirement: 87.0 [78.0–92.0], p < 0.001). To identify factors independently associated with fatigue severity, a multiple linear regression model was performed including variables showing significant univariate associations. Higher FSMCt was independently associated with greater disability (EDSS; β=1.79, 95%CI [0.31–3.27], p = 0.02), higher depressive symptom severity (BDI-II; β=0.77, 95%CI [0.53–1.03], p < 0.001), and early retirement (β=7.93, 95%CI [0.74–15.12], p = 0.03) (Figure 1(c); Table 3).

**Table 3.** Linear regression model for baseline data.

FSMC at baseline as dependent variable		
Variable	β (95% CI)	p-value
EDSS	1.79 (0.31–3.27)	0.02
BDI-II	0.77 (0.53–1.03)	<0.001
PPMS	2.62 (−6.22–11.47)	0.56
Early retirement	7.93 (0.74–15.12)	0.03
Smoking	1.44 (−3.84–6.72)	0.59

BDI-II, Beck Depression Inventory II; EDSS, Expanded Disability Status Scale; PPMS, primary progressive multiple sclerosis.

### Within-group changes in FSMC over 12 weeks

**Figure 2.**
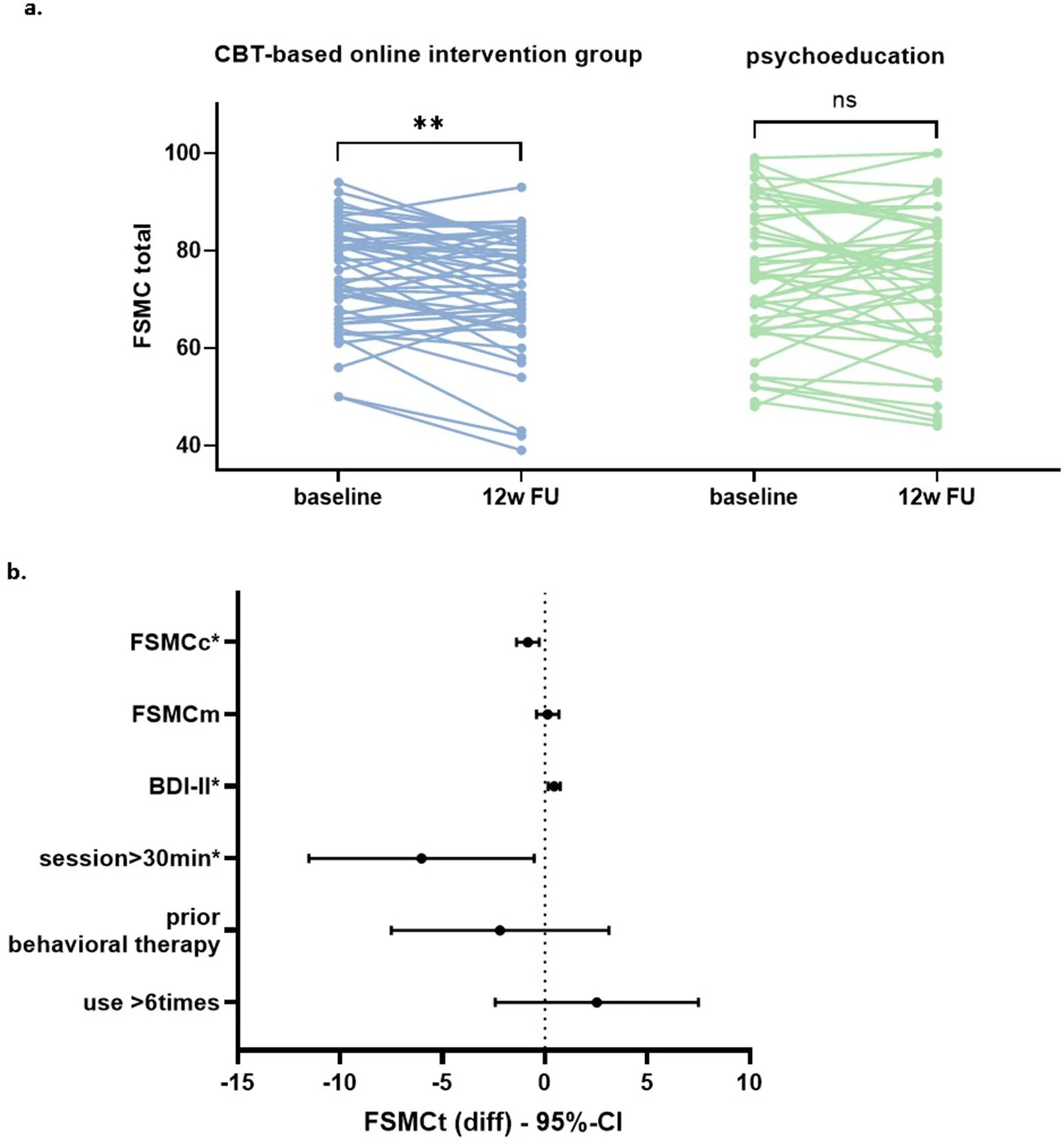
CBT-based online intervention has a positive effect on fatigue. (a) Considering only patients who completed the 12-week follow up (12w FU; CBT-based online intervention n = 49 and psychoeducation n = 50); a significant improvement in FSMC was obtained in patients using the CBT-based online intervention (repeated measures ANOVA: F(1,48) = 10.99, **: p = 0.002), but not in the psychoeducation group (repeated measures ANOVA: F(1,49) = 1.26, p = 0.27, ns: not significant). (b) Forest plot showing the determined regression coefficient for FSMC total (FSMCt) difference (defined as FSMCt at 12-week follow up minus FSMCt at baseline). The multiple linear regression model revealed FSMCc, BDI-II and sessions > 30 min as significant predictors for the dependent variable FSMCt difference. *12w FU, 12-week follow up;* *CI: confidence interval*; *FSMCc: Fatigue Scale for Motor and Cognitive Functions cognitive; FSMCm: Fatigue Scale for Motor and Cognitive Functions motoric; FSMCt: Fatigue Scale for Motor and Cognitive Functions total*.

A total of 99 patients (CBT-based online intervention group n = 49, psychoeducation n = 50) completed the observation period and answered the FSMC questionnaire 12 weeks (12w) after baseline (12wFSMCt). Fifteen study participants of the CBT-based online intervention group were lost either due to difficulties registering for the program (n = 6) or not registering at all (n = 9). In a within-group analysis, FSMCt decreased significantly from baseline to 12w in the CBT-based online intervention group (repeated measures ANOVA: F(1,48) = 10.99, p = 0.002), whereas no significant change was observed in the psychoeducation group (repeated measures ANOVA: F(1,49) = 1.26, p = 0.27) (Figure 2(a)).

To formally compare treatment effects between groups, an analysis of covariance was performed with 12wFSMCt as the outcome and baseline FSMC as a covariate. In this model, the adjusted between-group difference in 12wFSMCt (psychoeducation vs CBT-based online intervention) was 1.8 points (95%CI: [–2.0−5.6], p = 0.35), indicating no statistically significant between-group difference. In sensitivity analyses, comparable results were observed in the cross-over group, and when the CBT-based online intervention and crossover groups were analyzed as a pooled sample (Supplemental Figure 1+2). The CBT intervention was well tolerated, with no adverse events reported during the study period.

### Treatment effects on depressive and anxiety symptoms

At baseline, BDI-II and HADS were comparable between the CBT-based online intervention group and the psychoeducation group. In ANCOVA models adjusted for baseline symptom severity, participants receiving the CBT-based online intervention showed lower depressive symptom levels at 12w compared with the psychoeducation group, as reflected by significantly lower BDI-II scores (adjusted between-group difference: β=2.82, 95%CI: [0.14–5.50], p = 0.040). For anxiety and depressive symptoms assessed by HADS, no statistically significant between-group difference was observed at 12w after adjustment for baseline HADS score (adjusted between-group difference: β=1.23, 95%CI: [−1.07–3.52], p = 0.291). These analyses were specified as secondary and exploratory and were not adjusted for multiple comparisons.

### Exploratory predictors of improvement within the CBT-based intervention group

To explore which baseline characteristics were associated with greater improvement within the CBT-based online intervention group, we performed linear regression analyses using change in FSMCt (FSMCtdiff=12wFSMCt - BaselineFSMCt) as the dependent variable. These analyses were restricted to participants receiving the CBT-based intervention and should therefore be interpreted as hypothesis-generating only. A negative difference reflects an improvement in fatigue symptoms. Higher baseline FSMCc scores were associated with greater improvement (regression coefficient β=-0.84, 95%CI: [–1.39– –0.29], p = 0.004). In contrast, higher baseline BDI-II scores were associated with less improvement (β=0.45, 95%CI: [0.16–0.75], p = 0.004). Finally, ≥30 min usage per week was linked to improved fatigue outcomes (β=-6.03, 95%CI: [–11.53 − -0.532], p = 0.03) (Figure 2(c); Table 4).

**Table 4.** Multiple linear regression model for follow up data.

FSMCtdiff.as dependent variable		
Variable	β (95%CI)	p-value
FSMCc	−0.84 (−1.39- −0.29)	0.004
FSMCm	0.13 (−0.41–0.68)	0.623
BDI-II	0.45 (0.16–0.75)	0.004
Use > 6 times of online intervention	2.53 (−2.42–7.49)	0.306
Session of online intervention >30 min	−6.03 (−11.53- −0.53)	0.033
Prior behavioral therapy	−2.19 (−7.51–3.12)	0.408

BDI-II, Beck Depression Inventory II; FSMCc, Fatigue Scale for Motor and Cognitive Functions cognitive; FSMCm, Fatigue Scale for Motor and Cognitive Functions motor.

## Discussion

Fatigue remains one of the most common symptoms reported by pwMS. So far, pharmacological approaches are not specifically effective for MS-related fatigue. However, there is growing evidence supporting the effectiveness of CBT. The introduction of the CBT-based online intervention elevida, a DiGA, presented a new perspective on managing fatigue. This study aimed to assess the feasibility and effectiveness of such an intervention in a fatigue cohort in a clinical routine setting.

In a pragmatic real-world randomized setting, the CBT-based online intervention was associated with within-group improvements in fatigue, but did not demonstrate statistical superiority over psychoeducation in between-group analyses.

Fatigue and depressive symptoms were strongly associated at baseline, confirming their close clinical relationship in pwMS. Depressive symptoms assessed by BDI-II were significantly lower at 12 weeks in the CBT-based intervention group compared with psychoeducation; no between-group effect was observed for anxiety symptoms measured by HADS. This pattern suggests that the intervention may have a protective effect on depressive symptoms, which could indirectly influence fatigue perception.

Furthermore, higher FSMC scores were observed in smokers, potentially reflecting additional inflammatory burden, although smoking is also closely linked to socioeconomic and psychosocial factors that may contribute to fatigue. Higher fatigue levels were also associated with early retirement, underscoring the impact of fatigue on employment status in pwMS.

Within our study population, 76.9% of the CBT-based online intervention group completed the observational period, highlighting general feasibility and usability of DiGA in pwMS. Our findings extend those of Pöttgen et al.^18^ by demonstrating beneficial effects of a CBT-based online intervention on fatigue under real-world clinical conditions, while no significant fatigue reduction was observed in the psychoeducation group.

Exploratory within-group analyses suggested that greater fatigue improvement following the CBT-based online intervention was associated with higher baseline cognitive fatigue, lower baseline depressive symptom severity, and longer session duration, indicating engagement as a potentially relevant factor. This finding highlights the importance of recognizing and addressing depression as a potential comorbidity in need of specific treatment.

Our study is not without limitations. The sample size was moderate, recruitment was limited to a single center, and no a priori power calculation was performed, which may have limited the ability to detect small-to-moderate between-group differences. Fatigue outcomes were based on self-reported measures, which may be influenced by reporting bias. The psychoeducation control intervention was non-interactive, and engagement was not monitored, limiting its effectiveness and comparability. Although psychoeducation served as an active comparator, it may not fully control for non-specific effects such as expectancy or engagement. Follow-up was restricted to 12 weeks; longer-term effects remain unknown. Finally, the study was not prospectively registered, and no published protocol is available.

With an increasing number of digital tools becoming available, it is important to assess their practical applicability in clinical practice and to identify pwMS most likely to benefit. Based on our findings, CBT-based online interventions may be particularly suitable for pwMS with severe cognitive fatigue. DiGA may represent a complementary approach to established psychological care; however, our study was not designed to compare digital interventions with face-to-face psychotherapy. Moreover, assessing and adequately treating comorbid depression in pwMS is crucial. Our results highlight the importance of addressing fatigue not only in terms of symptom management but also regarding its social implications.

## Supplemental Material

sj-docx-1-mso-10.1177_20552173261468118 - Supplemental material for Real world observation of fatigue in multiple sclerosis comparing self-guided online cognitive behavioral therapy versus psychoeducation Supplemental material, sj-docx-1-mso-10.1177_20552173261468118 for Real world observation of fatigue in multiple sclerosis comparing self-guided online cognitive behavioral therapy versus psychoeducation by Maria Protopapa, Jana Samarin, Muriel Schraad, Livia S Lang, Marianne Hahn, Frauke Zipp and Stefan Bittner in Multiple Sclerosis Journal – Experimental, Translational and Clinical
